# The association of beliefs about medicines with medication adherence and glycemic control among patients with type 2 diabetes: a cross-sectional survey

**DOI:** 10.3389/fendo.2026.1691701

**Published:** 2026-02-10

**Authors:** Haiyan Li, Nannan Wang, David J. McIver, Lu Zhang, Junfei Shi, Dongwei Liu, Hui Min

**Affiliations:** 1 Department of Pharmacy, Xi’an People’s Hospital (Xi’an Fourth Hospital), Xi’an, China; 2 Department of Pharmacy, The Second Affiliated Hospital of Inner Mongolia Medical University, Hohhot, China; 3 Mclver Epi Scientific Consulting, Nanaimo, BC, Canada; 4 Department of Pharmacy, The First Affiliated Hospital of Xi’an Jiaotong University, Xi’an, China

**Keywords:** beliefs about medicines, China, glycemic control, hypoglycemic agents, medication adherence, nurses, pharmacists

## Abstract

**Background:**

Beliefs about medicines have been identified as important determinants of medication adherence for multiple chronic diseases such as hypertension, diabetes, and asthma. This study aimed to examine the association of beliefs about medicines with medication adherence and glycemic control among patients with type 2 diabetes (T2D) in Xi’an, China.

**Methods:**

A cross-sectional study was conducted with T2D patients recruited from two large tertiary hospitals in Xi’an, China, from August 1, 2024 to March 31, 2025. The Beliefs about Medicines Questionnaire (BMQ) was used to assess patients’ beliefs about hypoglycemic agents, and the Adherence to Refills and Medications Scale in Diabetes (ARMS-D) was used to assess adherence to hypoglycemic agents. The recommended cut-off of HbA1c<7.0% was used as an indicator of glycemic control. Logistic regression analyses were performed to investigate the determinants of both medication adherence and glycemic control. Receiver operating characteristic (ROC) analyses were conducted to assess the performance of the logistic regression model in predicting adherence to hypoglycemic agents.

**Results:**

Of these 446 patients enrolled in the study, the overall medication adherence was 32.5%, while 30.5% of patients achieved adequate glycemic control (HbA1c <7.0%). Multivariable logistic regression analyses revealed that patients who expressed stronger concern beliefs about the medicine had 1.20 (95% CI = 1.130-1.264) and 1.08 (95% CI = 1.022-1.136) times greater odds of non-adherence to hypoglycemic agents and inadequate glycemic control, respectively, than those expressed weaker concern beliefs, while necessity beliefs about the medicine were significantly negatively associated with medication non-adherence (adjusted OR 0.873 [0.814, 0.936]; p<0.001) and inadequate glycemic control (adjusted OR 0.916 [0.853, 0.983]; p = 0.015). This study found that presence of comorbidities and longer duration of diabetes (>10 years) was associated with medication adherence. Diabetes duration for 5–10 years and higher income were found to be associated with glycemic control, while taking injectable hypoglycemic agents and alcohol consumption were associated with inadequate glycemic control among T2D patients.

**Conclusion:**

This study revealed the status of suboptimal glycemic control and medication adherence of patients with T2D in Xi’an. Pharmacists and nurses could play an important role in educational and behavioral intervention of patients’ beliefs about medicines.

## Background

Diabetes is a major health issue that has reached alarming levels across the world. In 2024, it is estimated that 589 million adults (20–79 years) worldwide are living with diabetes, and this number is predicted to rise to 853 million by 2050 (1). China accounts for 1 in 4 of all adults living with diabetes worldwide (1). It is estimated that the prevalence of diabetes in China is around 13.8%, which is among the highest incidences of chronic disease (1, 2). Due to its large population and high prevalence of diabetes, China had approximately 148.0 million adults (aged 20–79 years) with diabetes in 2024; and the number of adults with diabetes is anticipated to rise to 168.3 million in 2050 (1). Diabetes imposes a substantial economic burden on the health systems in China (3), with total diabetes health expenditure of 169 billion (USD) in 2024 (1).

Type 2 diabetes (T2D) is the most common type of diabetes, accounting for nearly 90% of diabetes worldwide (4). The management of T2D include a healthy diet, physical activity, continuing medication, blood glucose monitoring and patient self-management education (4). The goals of diabetes treatment are to keep blood glucose levels as near to normal as possible, while avoiding acute and chronic complications (4, 5). It was reported that an intensive glucose-lowering strategy resulted in a 17% reduction in retinopathy, 18% reduction in macroalbuminuria, 32% reduction in end-stage renal disease, and 13% reduction in non-fatal myocardial infarction (6). However, most patients with T2D do not achieve sufficient glycemic control in China (7).

Medication adherence - the extent to which a person’s behavior of taking medication corresponds with agreed recommendations from a healthcare provider - is a key determinant in T2D treatment success (8). A systematic review illustrates that the self-reported medication adherence rate among diabetic patients ranges from 27.1% to 80.1% in China, with the range in reporting mainly attributable to the method used to measure adherence (9). Patients with T2D who are non-adherent to their maintenance medication may experience suboptimal glycemic control, increased risk of complications, hospitalization and mortality (10). Assessing patients’ glycemic control is part of the comprehensive management of patients with diabetes. Knowing the factors influencing medication adherence and glycemic control are important for clinical intervention and better treatment outcomes in patients with T2D (11).

Medication beliefs are an individual’s attitudes toward medication adherence’s benefits and potential harms (12). The Beliefs about Medicines Questionnaire (BMQ) assesses patients’ beliefs about medications prescribed for a particular illness. It consists of two scales: the Specific-Necessity subscale (a five-item scale) that assesses personal beliefs about the necessity of taking medication, and the Specific-Concerns subscale (a six-item scale), which focuses on the patients’ concerns of having potential adverse consequences of prescribed medication (13). Previous research has shown that for multiple chronic diseases, holding strong beliefs in the necessity of medication and weak concern beliefs were predictive of medication adherence (14, 15). Beliefs about medicines account for 19% of the variance in adherence to medication in chronic illness, which offered greater predictability than any other clinical or sociodemographic factors (13). Furthermore, the correlations between specific beliefs about medicines and medication adherence in the Chinese population seemed stronger than among the Western population (15).

The association between medication beliefs and medication adherence among patients with T2D has been repeatedly validated internationally (16–20), as well as in eastern and southern regions of China (21, 22). However, whether beliefs about medicines predict glycemic control is controversial (10, 18, 23). There is a significant research gap about the association of beliefs about medicines with medication adherence and glycemic control in patients with T2D in Northwestern China. Although the area of Xi’an accounted for only 5.23% of the total area of Shaanxi Province, its population accounted for 25.88%, with a population of approximately 13 million. As the largest city in Northwest China and the capital city of Shaanxi province, the area of Xi’an differed from the previously reported areas in terms of race, cultural background, and healthcare resources, and all of these factors may contribute to beliefs about medications, medication adherence and glycemic control in patients with T2D. We hypothesized that beliefs about medicines would be associated with medication adherence and glycemic control, and that holding strong beliefs in the necessity of medication, and weak concern beliefs, would be predictor of improved medication adherence and glycemic control. If this hypothesis is correct among this population, evidence-based interventions targeting patients’ beliefs about medicines may help improve medication adherence and glycemic control in our region. This study aimed to determine the association of beliefs about medicines with medication adherence and glycemic control among patients with T2D in the area of Xi’an.

## Methods

### Study subjects

A cross-sectional, questionnaire-based survey was conducted with 446 adult T2D patients recruited from two large tertiary hospitals in Xi’an City, Shaanxi Province of Northwestern China from August 1, 2024 to March 31, 2025. Participants were both outpatients and inpatients who visited the endocrine clinics of Xi’an People’s Hospital (Xi’an Fourth Hospital) and the First Affiliated Hospital of Xi’an Jiaotong University, two large tertiary teaching hospitals.

The inclusion criteria included patients who 1) were ≥18 years of age; 2) had an existing diagnosis of T2D; 3) had been treated with hypoglycemic agents for more than one year; 4) with the ability to record medicine information and monitor blood glucose levels; 5) had a telephone contact record in the electronic medical records; 6) agreed to participate in the survey. The exclusion criteria for this study were as follows: 1) without laboratory indicators, including glycosylated hemoglobin (HbA1c), fasting plasma glucose (FPG), and postprandial plasma glucose (PPG) in electronic medical records within 3 days of hospital admission; 2) patients had acute life-threatening conditions such as acute cerebral infarction, myocardial infarction or other critical illness; 3) pregnant women; 4) patients with malignancy; 5) dialysis patients; 6) those who could not communicate due to physical or mental problems. Eligible patients were identified by reviewing the electronic medical records of patients in the two hospitals, and investigators contacted eligible patients by phone to complete the survey.

### Sample size

The required sample size for the study was calculated by using the following formula: *n* = *z*^2^*p(1-p)/d*^2^, where *n* was the sample size, *z* was coefficient of confidence interval (1.96), *p* was the proportion of patients with medication adherence, and *d* was type I error level of 0.05. Generally, adherence to long-term therapy for chronic diseases in developed countries averages 50%, and the rates are even lower in developing countries (8). A minimum sample size of 384 patients was required based on the above assumptions. A total of 491 respondents agreed to participate in the survey. Forty-five questionnaires were not completed because the survey was interrupted by various reasons. A final sample size of 446 (90.8%) patients was recruited in this study.

### Survey procedures

The purpose and content of the study were explained to eligible participants and oral informed consent was obtained before they responded to the targeted questions. Patients were asked several questions via telephone, lasting about 15–20 minutes, and answers were documented. All the investigators had received standardized training on survey procedures and communication skills. The investigators followed a standardized template to guide the telephone conversation with the subjects.

### Measurement instruments

#### Beliefs about medicines questionnaire-specific

The BMQ-Specific developed by Horne et al. was used to assess patients’ beliefs about the medication prescribed for a particular illness (13). Respondents must indicate their degree of agreement with each individual statement of the 11 questions on a five-point Likert scale, ranging from 1 (strongly disagree) to 5 (strongly agree). Higher scores indicate stronger concern beliefs or necessity beliefs about hypoglycemic agents.

#### Adherence to refills and medications scale in diabetes

The ARMS-D is a validated 11-item scale used to measure adherence to taking and refilling medications among patients with diabetes (24, 25). The ARMS-D scale comprises two subscales: 7 items about adherence to taking medications and 4 items about refill prescriptions, respectively. Each of the 11 items was measured on a four-point Likert-type scale (1=none of the time, 2=some of the time, 3=most of the time, and 4=all of the time). Scoring ranges from 11 to 44 points, with lower scores representing better adherence. According to the published literature (24), patients were classified into two groups based on their total adherence scores: patients who had a total score of 11 points were labeled as adherent group, while others (above 11 points) were considered as nonadherent group. The Chinese versions of the ARMS-D and BMQ-Specific scales were adapted for use in our study after we obtained authorization from the developers of the scales (see **Supplementary Files 1, 2**).

### Data collection

Sociodemographic characteristics, diseases related characteristics, hypoglycemic agents related characteristics, laboratory indicators and lifestyle-related characteristics were obtained from medical records or telephone conversations. Description of variables included in the survey was shown in **Supplementary Table S1** (see **Supplementary File 3**). BMI (kg/m^2^) is calculated as weight in kilograms divided by height in meters squared. Income is distinguished based on a defined threshold of 4000 yuan (CNY) per month. HbA1c was used to determine how well patients controlled their glucose level over the prior 3 months. Patients’ laboratory indicators including HbA1c (%), FPG (mmol/L), and PPG (mmol/L) were extracted from electronic medical records within 3 days of hospital admission. The questionnaire adopted in our study was provided as a supplementary file (see **Supplementary File 4**).

### Outcome measurements

The recommended cut-off of HbA1c<7.0% was used as an indicator of glycemic control among patients with diabetes who were treated (4, 7, 26). Patients with HbA1c value above 7.0% were assigned to inadequate glycemic control group, whereas less than 7.0% were assigned to adequate glycemic control group. The prevalence of non-adherence to hypoglycemic agents, as well as the proportion of individuals with inadequate glycemic control were investigated as the primary outcome. The association of beliefs about medicines with medication adherence and glycemic control among patients with T2D who were treated were investigated as secondary outcome.

### Statistical analysis

Data from the survey were descriptively presented, where categorical variables were presented as counts and percentages and continuous variables as means and standard deviations (SD). Differences in the candidate variables between adherent and non-adherent groups, as well as adequate and inadequate glycemic control groups were evaluated using the Chi-square test for categorical variables, the Mann-Whitney test for non-normally distributed continuous variables, and the independent samples t-test for normal continuous variables. Univariable and multivariable (all the variables were included) logistic regression models were used to characterize the determinants of medication non-adherence and inadequate glycemic control. In the adjusted logistic regression model, we adjusted for all covariates using the backward elimination. Receiver operator characteristic (ROC) analyses were conducted to assess the performance of the logistic regression model in predicting medication non-adherence. All analysis was performed by using the SPSS v25.0 Statistical Software Package for Windows. A p value <0.05 was considered statistically significant for all analysis.

The questionnaire has demonstrated good reliability and validity. The internal reliability of the entire questionnaire measured by Cronbach’s alpha value was 0.73, which means an acceptable level of reliability in this study. We performed Confirmatory Factor Analysis (CFA) using R, the Root Mean Square Error of Approximation (RMSEA) were 0.078, indicating favorable validity.

## Results

### Patient characteristics

During the study period, 446 patients were enrolled, of which 275 (61.7%) were male. The mean age of the participants was 56.1 ± 12.1 years. The majority (70.7%) had been diagnosed with T2D for less than or equal to 10 years, while a minority (29.4%) had been diagnosed with T2D for more than 10 years. Oral hypoglycemic agents were taken by 42.4% and injectable hypoglycemic agents (on their own or combined with oral hypoglycemic agents) by 57.6% of the study population. More than half of participants (58.7%) reported that they had diabetic complications. The majority (87.4%) patients regularly monitored their blood glucose by themselves, while 228 (51.1%) patients exercised more than three times per week. The majority of patients (52.7%) in the present study expressed strong concerns about the potential negative effects of hypoglycemic agents, while less than half of the patients (49.1%) expressed strong beliefs about the necessity of medications. The demographic and clinical characteristics of the study subjects by medication adherence are presented in **Table 1**.

**Table 1:** Demographic and clinical characteristics of the study subjects by medication adherence.

Characteristics	Total (n=446, %)	Adherent (n=145, %)	Nonadherent (n=301, %)	*P-value*
**Age (means ± SD)**	56.06 ± 12.10	57.96 ± 11.83	55.15 ± 12.14	**0.021**
**Age (years)**				0.209
18-44	80 (17.9%)	20 (25.0%)	60 (75.0%)	
45-64	244 (54.7%)	80 (32.8%)	164 (67.2%)	
≥65	122 (27.4%)	45 (36.9%)	77 (63.1%)	
**Gender**				0.770
Female	171 (38.3%)	57 (33.3%)	114 (66.7%)	
Male	275 (61.7%)	88 (32.0%)	187 (68.0%)	
**BMI (means ± SD)**	23.91 ± 3.39	23.87 ± 3.39	23.93 ± 3.40	0.858
**BMI (kg/m^2^)**				0.995
≤23.9	246 (55.2%)	80 (32.5%)	166 (67.5%)	
24-27.9	153 (34.3%)	50 (32.7%)	103 (67.3%)	
≥28	47 (10.5%)	15 (31.9%)	32 (68.1%)	
**Smoking status**				0.187
No	257 (57.6%)	90 (35.0%)	167 (65.0%)	
Yes	189 (42.4%)	55 (29.1%)	134 (70.9%)	
**Alcohol consumption**				0.727
No	285 (63.9%)	91 (31.9%)	194 (68.1%)	
Yes	161 (36.1%)	54 (33.5%)	107 (66.5%)	
**Education level**				**0.031**
≤High school	257 (57.6%)	73 (28.4%)	184 (71.6%)	
>High school	189 (42.4%)	72 (38.1%)	117 (61.9%)	
**Employment status**				0.745
Not employed	272 (61.0%)	90 (33.1%)	182 (66.9%)	
Employed	174 (39.0%)	55 (31.6%)	119 (68.4%)	
**Income/month**				0.283
Low	207 (46.4%)	62 (30.0%)	145 (70.0%)	
High	239 (53.6%)	83 (34.7%)	156 (65.3%)	
**Duration of diabetes (means ± SD)**	8.03 ± 6.53	9.70 ± 7.40	7.23 ± 5.91	**0.001**
**Duration of diabetes**				**0.005**
<5 years	172 (38.6%)	43 (25.0%)	129 (75.0%)	
5–10 years	143 (32.1%)	46 (32.2%)	97 (67.8%)	
>10 years	131 (29.4%)	56 (42.7%)	75 (57.3%)	
**Presence of other chronic diseases**				0.177
No	139 (31.2%)	39 (28.1%)	100 (71.9%)	
Yes	307 (68.8%)	106 (34.5%)	201 (65.5%)	
**Presence of diabetic complications**				0.070
No	184 (41.3%)	51 (27.7%)	133 (72.3%)	
Yes	262 (58.7%)	94 (35.9%)	168 (64.1%)	
**Hypoglycemic agents prescribed**				0.136
Oral	189 (42.4%)	53 (28.0%)	136 (72.0%)	
Injectable	29 (6.5%)	8 (27.6%)	21 (72.4%)	
Oral and injectable	228 (51.1%)	84 (36.8%)	144 (63.2%)	
**Medications prescribed for other chronic diseases**				
Antihypertensives	199 (44.6%)	64 (32.2%)	135 (67.8%)	0.887
Antiplatelets	125 (28.0%)	49 (39.2%)	76 (60.8%)	0.060
Statin	261 (58.5%)	95 (36.4%)	166 (63.6%)	**0.037**
**Self-monitoring of blood glucose**				0.713
No	56 (12.6%)	17 (30.4%)	39 (69.6%)	
Yes	390 (87.4%)	128 (32.8%)	262 (67.2%)	
**Beliefs about medicines (mean score ± SD)**	35.23 ± 5.27	34.26 ± 5.23	35.70 ± 5.24	**0.006**
**Necessity beliefs (mean score ± SD)**	18.15 ± 3.25	19.01 ± 3.42	17.74 ± 3.10	**<0.001**
**Concern beliefs (mean score ± SD)**	17.08 ± 4.15	15.25 ± 4.62	17.96 ± 3.60	**<0.001**
**Exercise per week**				0.404
≥3times	228 (51.1%)	70 (30.7%)	158 (69.3%)	
<3times	218 (48.9%)	75 (34.4%)	143 (65.6%)	

Categorical variables were compared using the Chi-square test, while non-normally distributed continuous variables were analyzed using the Mann-Whitney test and normal continuous variables were analyzed using the independent samples t-test. Bold values indicated statistical significance (p -value<0.05).

The overall medication adherence was 32.5%. The averaged medication adherence levels of the 446 participants were 17.71 ± 4.53. Only 136 (30.5%) patients achieved the target glycemic level (<7.0%) in this study. The averaged HbA1c levels of the 446 participants were 8.7 ± 2.2%, the averaged FBG were 9.0 ± 3.0 mmol/L, and the averaged PPG were 14.4 ± 5.1mmol/L, respectively. The demographic and clinical characteristics of the study subjects by glycemic control are presented in **Table 2**.

**Table 2:** Demographic and clinical characteristics of the study subjects by glycemic control.

Characteristics	Total (n=446, %)	Adequate glycemic control (n=136, %)	Inadequate glycemic control (n=310, %)	*P-value*
**Age (means ± SD)**	56.06 ± 12.10	58.18 ± 10.70	55.15 ± 12.57	**0.009**
**Age (years)**				0.079
18-44	80 (17.9%)	16 (20.0%)	64 (80.0%)	
45-64	244 (54.7%)	80 (32.8%)	164 (67.2%)	
≥65	122 (27.4%)	40 (32.8%)	82 (67.2%)	
**Gender**				0.277
Female	171 (38.3%)	47 (27.5%)	124 (72.5%)	
Male	275 (61.7%)	89 (32.4%)	186 (67.6%)	
**BMI (means ± SD)**	23.91 ± 3.39	23.99 ± 2.90	23.88 ± 3.59	0.718
**BMI (kg/m^2^)**				0.979
≤23.9	246 (55.2%)	76 (30.9%)	170 (69.1%)	
24-27.9	153 (34.3%)	46 (30.1%)	107 (69.9%)	
≥28	47 (10.5%)	14 (29.8%)	33 (70.2%)	
**Smoking status**				0.335
No	257 (57.6%)	83 (61.0%)	174 (56.1%)	
Yes	189 (42.4%)	53 (39.0%)	136 (43.9%)	
**Alcohol consumption**				**0.018**
No	285 (63.9%)	98 (72.1%)	187 (60.3%)	
Yes	161 (36.1%)	38 (27.9%)	123 (39.7%)	
**Education level**				0.185
≤High school	257 (57.6%)	72 (28.0%)	185 (72.0%)	
>High school	189 (42.4%)	64 (33.9%)	125 (66.1%)	
**Employment status**				0.823
Not employed	272 (61.0%)	84 (30.9%)	188 (69.1%)	
Employed	174 (39.0%)	52 (29.9%)	122 (70.1%)	
**Income/month**				0.094
Low	207 (46.4%)	55 (26.6%)	152 (73.4%)	
High	239 (53.6%)	81 (33.9%)	158 (66.1%)	
**Duration of diabetes** **(means ± SD)**	8.03 ± 6.53	7.85 ± 5.56	8.11 ± 6.91	0.665
**Duration of diabetes**				**0.003**
<5 years	172 (38.6%)	44 (25.6%)	128 (74.4%)	
5–10 years	143 (32.1%)	59 (41.3%)	84 (58.7%)	
>10 years	131 (29.4%)	33 (25.2%)	98 (74.8%)	
**Presence of other chronic diseases**				**0.011**
No	139 (31.2%)	31 (22.3%)	108 (77.7%)	
Yes	307 (68.8%)	105 (34.2%)	202 (65.8%)	
**Presence of diabetic complications**				0.202
No	184 (41.3%)	50 (27.2%)	134 (72.8%)	
Yes	262 (58.7%)	86 (32.8%)	176 (67.2%)	
**Hypoglycemic agents prescribed**				**<0.001**
Oral	189 (42.4%)	82 (43.4%)	107 (56.6%)	
Injectable	29 (6.5%)	3 (10.3%)	26 (89.7%)	
Oral and injectable	228 (51.1%)	51 (22.4%)	177 (77.6%)	
**Medications prescribed for other chronic diseases**				
Antihypertensives	199 (44.6%)	75 (37.7%)	124 (62.3%)	**0.003**
Antiplatelets	125 (28.0%)	45 (36.0%)	80 (64.0%)	0.115
Statin	261 (58.5%)	81 (31.0%)	180 (69.0%)	0.768
**Self-monitoring of blood glucose**				0.206
No	56 (12.6%)	13 (23.2%)	43 (76.8%)	
Yes	390 (87.4%)	123 (31.5%)	267 (68.5%)	
**Beliefs about medicines** **(mean score ± SD)**	35.23 ± 5.27	34.83 ± 5.35	35.41 ± 5.24	0.286
**Necessity beliefs** **(mean score ± SD)**	18.15 ± 3.25	18.78 ± 3.36	17.88 ± 3.18	**0.007**
**Concern beliefs** **(mean score ± SD)**	17.08 ± 4.15	16.05 ± 4.51	17.53 ± 3.91	**0.001**
**Exercise per week**				0.183
≥3times	228 (51.1%)	76 (55.9%)	152 (49.0%)	
<3times	218 (48.9%)	60 (44.1%)	158 (51.0%)	
**Medicine adherence** **(mean score ± SD)**	17.71 ± 4.53	17.21 ± 4.26	17.92 ± 4.63	0.128
**Medicine adherence**				0.406
Adherence	145 (32.5%)	48 (35.3%)	97 (31.3%)	
Non-adherence	301 (67.5%)	88 (64.7%)	213 (68.7%)	

Categorical variables were compared using the Chi-square test, while non-normally distributed continuous variables were analyzed using the Mann-Whitney test and normal continuous variables were analyzed using the independent samples t-test. Bold values indicated statistical significance (p-value<0.05).

### Predictors of medication non-adherence

Univariable and multivariable logistic regression analyses of determinants of non-adherence to hypoglycemic agents were provided in **Table 3**.

**Table 3:** Univariable and multivariable logistic regression analyses of determinants of non-adherence to hypoglycemic agents.

Characteristics	Unadjusted OR (95% CI)	*P-value*	Adjusted OR (95% CI)	*P-value*
Age (years)				
18-44	1.000 (Reference)			
45-64	0.683 (0.386, 1.211)	0.192		
≥65	0.570 (0.305, 1.066)	0.079		
Gender				
Female	1.000 (Reference)			
Male	1.062 (0.708, 1.595)	0.770		
BMI (kg/m^2^)				
≤23.9	1.000 (Reference)			
24-27.9	0.993 (0.646, 1.527)	0.974		
≥28	1.028 (0.527, 2.007)	0.935		
Smoking status				
No	1.000 (Reference)			
Yes	1.313 (0.876, 1.969)	0.188		
Alcohol consumption				
No	1.000 (Reference)			
Yes	0.929 (0.616, 1.402)	0.727		
Education level				
≤High school	1.000 (Reference)			
>High school	0.645 (0.432, 0.961)	**0.031**		
Employment status				
Not employed	1.000 (Reference)			
Employed	1.070 (0.712, 1.608)	0.745		
Income/month				
Low	1.000 (Reference)			
High	0.804 (0.539, 1.198)	0.283		
Duration of diabetes				
<5 years	1.000 (Reference)		1.000 (Reference)	
5–10 years	0.703 (0.430, 1.150)	0.160	0.910 (0.523, 1.582)	0.738
>10 years	0.446 (0.274, 0.728)	**0.001**	0.438 (0.257, 0.746)	**0.002**
Presence of other chronic diseases				
No	1.000 (Reference)		1.000 (Reference)	
Yes	0.740 (0.477, 1.147)	0.177	0.558 (0.318, 0.977)	**0.041**
Presence of diabetic complications				
No	1.000 (Reference)			
Yes	0.685 (0.455, 1.032)	0.071		
Hypoglycemic agents prescribed				
Oral	1.000 (Reference)			
Injectable	1.023 (0.427, 2.451)	0.959		
Oral and injectable	0.668 (0.441, 1.013)	0.057		
Medications prescribed for other chronic diseases				
Antihypertensives	1.029 (0.691, 1.534)	0.887		
Antiplatelets	0.662 (0.430, 1.019)	0.061		
Statins	0.647 (0.429, 0.976)	**0.038**		
Self-monitoring of blood glucose				
No	1.000 (Reference)			
Yes	0.892 (0.486, 1.638)	0.713		
**Necessity beliefs**	0.877 (0.820, 0.939)	**<0.001**	0.873 (0.814, 0.936)	**<0.001**
**Concern beliefs**	1.181 (1.120, 1.245)	**<0.001**	1.195 (1.130, 1.264)	**<0.001**

Bold values indicated statistical significance (p-value<0.05). OR, odds ratio; CI, confidence interval. Patients who expressed stronger concern beliefs about the medicine was significantly associated with non-adherence to hypoglycemic agents among T2D patients.

In the univariable analysis, patients who expressed stronger concern beliefs about hypoglycemic agents (p<0.001) were significantly positively associated with medication non-adherence, while patients who expressed stronger necessity beliefs about hypoglycemic agents (p<0.001), received a high school diploma above (p = 0.031), were prescribed statins (p = 0.038), and with longer duration of diabetes (>10 years) (p = 0.001) were more likely to be adherent. Patients who expressed stronger concern beliefs about hypoglycemic agents were more likely to be nonadherent compared with those expressed weaker concern beliefs (unadjusted OR 1.181[1.120-1.245]). Patients who expressed stronger necessity beliefs about hypoglycemic agents were more likely to be adherent compared with those expressed weaker necessity beliefs (unadjusted OR 0.877 [0.820-0.939]). Patients who were prescribed statins were more likely to be adherent (unadjusted OR 0.647 [0.429-0.976]). Patients who received a high school diploma above were more likely to be adherent compared with those who received a high school diploma or below (unadjusted OR 0.645 [0.432-0.961]). Multivariable logistic regression analyses revealed that patients who expressed stronger concern beliefs about the medicine had 1.20 (95% CI = 1.130-1.264) times greater odds of non-adherence to hypoglycemic agents than those expressed weaker concern beliefs, while necessity beliefs (adjusted OR 0.873 [0.814, 0.936]; p<0.001) were significantly negatively associated with non-adherence to hypoglycemic agents in T2D patients. This study found that presence of comorbidities (adjusted OR 0.558 [0.318, 0.977]; p = 0.041) and longer duration of diabetes (>10 years) (adjusted OR 0.438 [0.257, 0.746]; p = 0.002) was associated with medication adherence.

**Figure 1:**
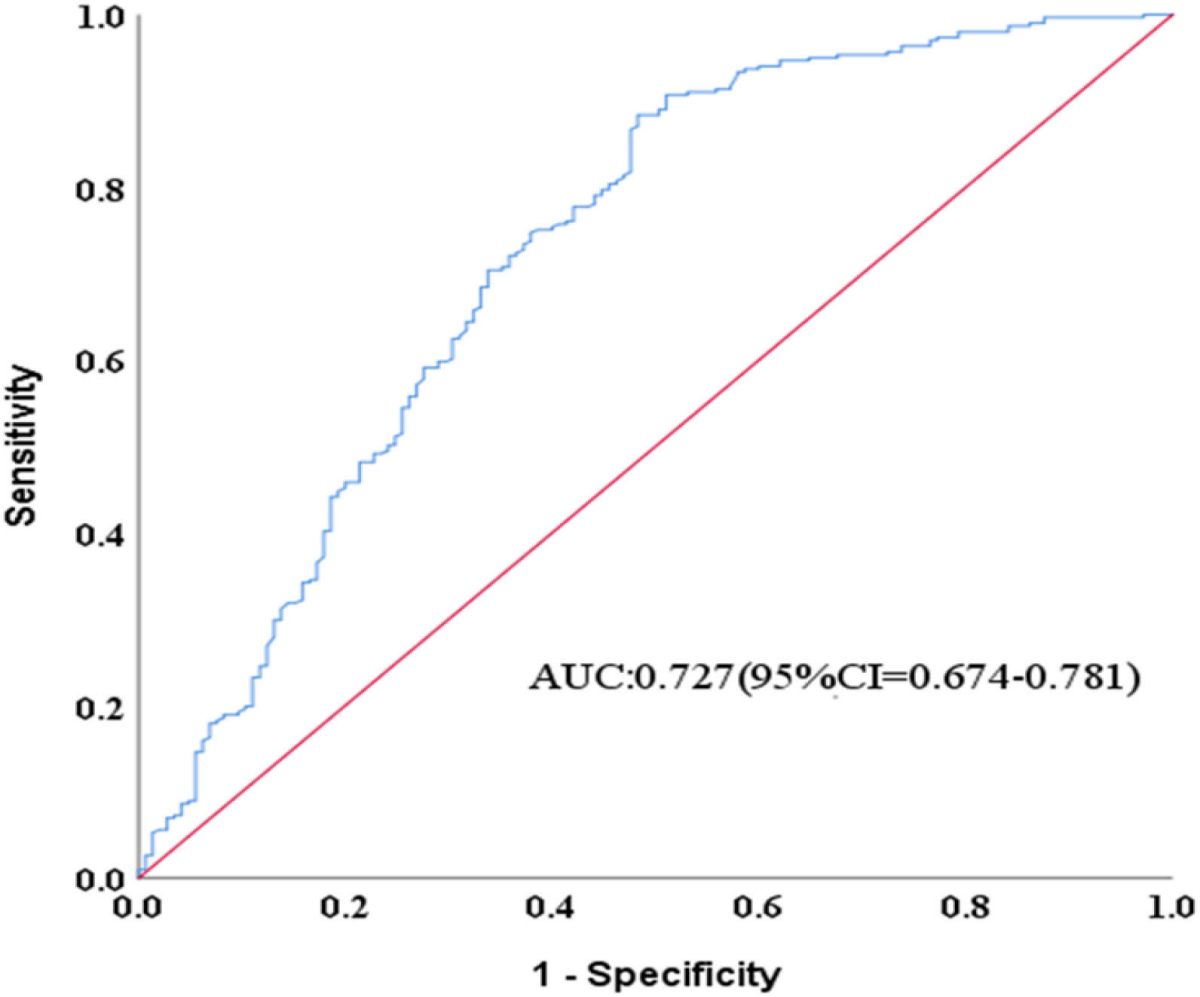
Receiver operating characteristic (ROC) curve for logistic regression model predicting non-adherence to antidiabetic agents: AUC of ROC curve = 0.727 (95% CI = 0.674-0.781). The ROC curve generated using SPSS v25.0 for a multivariable model predicting medication non-adherence.

The receiver operating characteristic (ROC) curve for logistic regression model predicting non-adherence to hypoglycemic agents is shown in **Figure 1**. The model provided an area under the curve (AUC) for the ROC curve of 0.73 (95% CI = 0.68-0.78).

### Predictors of inadequate glycemic control

Univariable and multivariable logistic regression analyses of determinants of inadequate glycemic control were provided in **Table 4**.

**Table 4:** Univariable and multivariable logistic regression analyses of determinants of inadequate glycemic control. .

Characteristics	Unadjusted OR (95% CI)	*P-value*	Adjusted OR (95% CI)	*P-value*
Age (years)				
18-44	1.000 (Reference)			
45-64	0.513 (0.279, 0.943)	**0.032**		
≥65	0.513 (0.263, 0.997)	**0.049**		
Gender				
Female	1.000 (Reference)			
Male	0.792 (0.520,1.206)	0.277		
BMI (kg/m^2^)				
≤23.9	1.000 (Reference)			
24-27.9	1.040 (0.671,1.613)	0.861		
≥28	1.054 (0.533,2.082)	0.880		
Smoking status				
No	1.000 (Reference)			
Yes	1.224 (0.811, 1.847)	0.335		
Alcohol consumption				
No	1.000 (Reference)		1.000 (Reference)	
Yes	1.696 (1.094, 2.629)	**0.018**	1.998 (1.167, 3.420)	**0.012**
Education level				
≤High school	1.000 (Reference)			
>High school	0.760 (0.506,1.141)	0.186		
Employment status				
Not employed	1.000 (Reference)			
Employed	1.048 (0.693,1.586)	0.823		
Income/month				
Low	1.000 (Reference)		1.000 (Reference)	
High	0.706 (0.469,1.062)	0.095	0.596 (0.368, 0.966)	**0.036**
Duration of diabetes				
<5 years	1.000 (Reference)		1.000 (Reference)	
5–10 years	0.489 (0.304,0.789)	**0.003**	0.502 (0.292, 0.861)	**0.012**
>10 years	1.021 (0.605,1.721)	0.938	0.729 (0.401, 1.324)	0.298
Presence of other chronic diseases				
No	1.000 (Reference)			
Yes	0.552 (0.347,0.878)	**0.012**		
Presence of diabetic complications				
No	1.000 (Reference)			
Yes	0.764 (0.504,1.156)	0.202		
Hypoglycemic agents prescribed				
Oral	1.000 (Reference)		1.000 (Reference)	
Injectable	6.642 (1.943,22.705)	**0.003**	6.032 (1.644, 22.130)	**0.007**
Oral and injectable	2.660 (1.741,4.064)	**<0.001**	3.175 (1.969, 5.120)	**<0.001**
Medications prescribed for other chronic diseases				
Antihypertensives	0.542 (0.361,0.815)	**0.003**		
Antiplatelets	0.703 (0.454,1.091)	0.116		
Statins	0.949 (0.624,1.417)	0.768		
Self-monitoring of blood glucose				
No	1.000 (Reference)			
Yes	0.656 (0.341,1.265)	0.208		
**Necessity beliefs**	0.914 (0.855,0.976)	**0.008**	0.916 (0.853, 0.983)	**0.015**
**Concern beliefs**	1.089 (1.037,1.144)	**0.001**	1.077 (1.022, 1.136)	**0.006**
Exercise per week				
≥3times	1.000 (Reference)			
<3times	0.759 (0.506, 1.139)	0.183		
Medicine adherence				
Adherence	1.000 (Reference)			
Non-adherence	1.198 (0.782,1.834)	0.406		

Bold values indicated statistical significance (p-value<0.05). OR, odds ratio; CI, confidence interval. Patients who expressed stronger concern beliefs about the medicine, the use of injectable hypoglycemic agents and alcohol consumption were significantly associated with inadequate glycemic control among T2D patients.

In the univariable analysis, inadequate glycemic control was significantly associated with older than 45 years (age between 45 and 64 years, p = 0.032 and older than 65 years, p = 0.049), alcohol consumption (p = 0.018), diabetes duration for 5–10 years (p = 0.003), presence of other chronic diseases (p = 0.012), taking injectable hypoglycemic agents (on their own (p = 0.003) or combined with oral hypoglycemic agents (p<0.001)), the concurrent use of antihypertensives (p = 0.003), necessity beliefs (p = 0.008) and concern beliefs (p = 0.001). Patients who were older than 45 years were associated with lower odds of inadequate glycemic control compared with those younger than 45 years (age between 45 and 64 years, unadjusted OR 0.513 [0.279, 0.943]; older than 65 years, unadjusted OR 0.0.513 [0.263, 0.997]). Compared with patients who didn’t drink alcohol, patients who drank alcohol were more likely to report inadequate glycemic control (unadjusted OR 1.696 [1.094, 2.629]). Compared with patients with less than 5 years duration of diabetes since diagnosis, those with diabetes duration for 5–10 years were less likely to report inadequate glycemic control (unadjusted OR 0.489 [0.304, 0.789]). Patients who were prescribed injectable hypoglycemic agents only (unadjusted OR 6.642 [1.943, 22.705]) or combined with oral agents (unadjusted OR 2.660 [1.741, 4.064]) were more likely to report inadequate glycemic control than those prescribed oral hypoglycemic agents only. Patients who expressed stronger concern beliefs about hypoglycemic agents were more likely to report inadequate glycemic control compared with those with weaker concern beliefs (unadjusted OR 1.089 [1.037, 1.144]). Patients who expressed stronger necessity beliefs about hypoglycemic agents indicated lower odds of inadequate glycemic control compared with those with weaker necessity beliefs (unadjusted OR 0.914 [0.855-0.976]).

Multivariable logistic regression analyses revealed that patients who expressed stronger concern beliefs about hypoglycemic agents had 1.08 (95% CI = 1.022-1.136) times greater odds of inadequate glycemic control than those expressed weaker concern beliefs, while necessity beliefs were significantly negatively associated with inadequate glycemic control (adjusted OR 0.916 [0.853, 0.983]; p=0.015). We also found that patients who were taking injectable hypoglycemic agents had more than six (95% CI = 1.644-22.130) (injectable hypoglycemic agents only) and three (95% CI = 1.969-5.120) times (injectable combined with oral hypoglycemic agents) greater odds of inadequate glycemic control, respectively, than those were taking oral hypoglycemic agents only. Patients who drank alcohol had more than two times (95% CI = 1.167-3.420) greater odds of inadequate glycemic control than those who didn’t drink alcohol. Patients with diabetes duration for 5–10 years (adjusted OR 0.502 [0.292, 0.861]; p=0.012) and higher income (adjusted OR 0.596[0.368, 0.966]; p=0.036) were significantly negatively associated with inadequate glycemic control.

## Discussion

This study identified the prevalence of non-adherence to hypoglycemic agents, as well as the proportion of individuals with inadequate glycemic control among patients with T2D who were treated in two large tertiary hospitals in Xi’an, China. It is noteworthy that medication adherence among T2D patients in Xi’an was suboptimal (32.5%) and relatively lower than previous findings reported from Singapore (40.2%) (27), Cameroon (45.6%) (28), Palestine (57.9%) (18), South Africa (67.0%) (29), North India (79.5%) (30) and Ghana (84.5%) (31). A systematic review by Krass et al. reported that medication adherence among diabetic patients ranged from 38.5% to 93.1% (32). A systematic review indicated that the prevalence of self-reported medication adherence among diabetic patients are 27.1% (Beijing City), 45.4% (Changzhou City), 59.0% (four cities in China), 80.1% (Shandong Province) in China (9). It was reported that only 14.5% of T2D patients discharged after receiving inpatient treatment at a tertiary hospital in Dongguan City, the Southern China, showed good adherence (22). The variation of adherence level between studies can be partially explained by differences in sample size, study design, methodologies (i.e., questionnaires) used for adherence measurements, health care settings, and regional distribution (19). Attaining optimal glycemic control has been deemed critical for achieving optimal health outcomes in the treatment of diabetes mellitus. This study demonstrated that a significant proportion of the patients with T2D who were treated were far from the target glycemic level. Only 30.5% of these patients achieved adequate glycemic control in this study, which was lower than that of patients in Guangzhou (35%) (33), the data from 161 hospitals across 18 provinces China (44.04%) (34), as well as the country as a whole (39.7%) (7). The adequate glycemic control rate in the present study appeared to be lower than studies reported in Jordan (41.5%) (23) and the United States (55.8%) (35), but was better than in studies conducted in Iraq (13.8%) (36) and Saudi Arabia (24.1%) (37).

This study identified positive association between medication beliefs and medication adherence in our region, which indicated that patients with stronger concern beliefs about the medicines tended to exhibit better medication non-adherence, while patients with stronger necessity beliefs about the medicines tended to exhibit better medication adherence. Numerous studies both internationally (10, 19, 20, 38) and domestically (21, 22) has demonstrated that patient’s perceived need for the prescribed medication and concerns about the potential negative effects of medicines were associated with medication adherence, which were consistent with the findings in this study. Horne and other researchers pointed out that patients who expressed stronger beliefs about the necessity of medications, the greater the perceived benefits of medication and the greater the likelihood that the individual would adopt adherence behaviors. In contrast, patients who expressed stronger beliefs about the concern of medications, the more the individual worries about the potential negative effects of medicines, the more barriers were perceived during medication administration, and consequently, the emergence of low adherence behaviors (13). Of the research exploring the association between medication beliefs and medication adherence, some demonstrated only necessity beliefs about the medicines were associated with medication adherence (39), while others had demonstrated that only concern beliefs about the medicines to be associated with medication adherence (16, 17). We found that concern beliefs about the medicines were positively associated with inadequate glycemic control, while necessity beliefs about the medicines were negatively associated with inadequate glycemic control. Beliefs about the necessity of medications were significantly associated with improved glycemic control in the study conducted in Jordan (23), which was consistent with our study. Beliefs about medicines are associated with glycemic control in studies of some, but not all populations (10, 18). As the modifiable aspects, healthcare professionals should focus on emphasizing medication necessity and overcoming medication concerns to improve medication adherence and hence to achieve a better glycemic control among patients with T2D who were treated in our region.

This study demonstrated that the presence of comorbidities was significantly associated with medication adherence among T2D patients, but not glycemic control. The findings regarding the association between comorbidities and medication adherence are currently mixed in international literature. The finding in the present study supported previous research suggesting that diabetes patients without concomitant comorbidities were more likely to discontinue their medication regimen (40). This result indicated that patients without comorbidities perceived their need to take medication was lower than those with increased comorbidities. Contrary findings have been reported, wherein it was reported that comorbid conditions had no relation to medication adherence (30), and patients with increased comorbidities are at risk of insulin non-persistence (41). Furthermore, it was reported that the presence of comorbidities was the determinant of poor glycemic control (11). Other studies demonstrated that the presence of comorbidities was associated with a decreased risk of poor glycemic control (36). The association of comorbidities with medication adherence and glycemic control still needs to be further explored in the future.

Patients who took injectable hypoglycemic agents (on their own or combined with oral hypoglycemic agents) had more than six (95% CI = 1.644-22.130) and three (95% CI = 1.969-5.120) times greater odds of inadequate glycemic control, respectively, than those who took oral hypoglycemic agents only. Our results were consistent with previous studies that indicated using injectable hypoglycemic agents (insulin or glucagon-like peptide-1 (GLP-1) receptor agonists) were independent risk factors for inadequate glycemic control (37). It was reported that taking insulin alone or combined with oral hypoglycemic agents to manage diabetes were associated with inadequate glycemic control (34, 36, 42). Due to the progressive nature of this disease, injectable hypoglycemic treatments may be added on to oral hypoglycemic to manage hyperglycemia effectively. It is worth noting that the progression of the disease could contribute to inadequate glycemic control status among injectable hypoglycemic medications users (36, 37).

We found alcohol consumption was associated with inadequate glycemic control. This finding supported previous research suggesting that alcohol intake had positive impact on inadequate glycemic control for diabetes (43–45). Alcohol influences glucose metabolism in several ways. It inhibits both glucose metabolism and glycogenolysis (45). Drinking alcohol can fluctuate the blood glucose level. A healthier lifestyle was significantly associated with adequate glycemic control in patients with T2D, and the recommendation of drinking alcohol for patients with diabetics should be cautious (44) Few studies have examined the association between income and glycemic control in patients with T2D. Our findings supported the previous study that demonstrated higher income was associated with improvement in glycemic control (45, 46). Previous studies revealed the association of low income and low adherence to medications with inadequate glycemic control (37). Low income decreases the likelihood of adherence to lifestyle modifications and treatment regimen, which ultimately leading to poor glycemic control.

The present study found that longer duration of diabetes (>10 years) was associated with medication adherence, and diabetes duration for 5–10 years was associated with glycemic control among T2D patients. It was reported that medication adherence increased with longer duration of disease, while glycemic control became worse (46, 47). The odds of medication adherence increased with longer duration of disease (>10 years) (30), which was consistent with our study. The possible reason may be that patients with longer duration of disease are more likely to interact better with the healthcare providers, understand their treatment regimen, and become more aware of their diseases, ultimately increasing medication adherence (30). It was reported that diabetes treatment for 5–10 years was found to be independent predictor of glycemic control among T2D patients (47, 48). However, several previous studies have demonstrated that T2D duration ≥5 years was associated with poor glycemic control (34, 47), which was inconsistent with our study. Other studies demonstrated that longer duration of diabetes (>10 years) was independent risk factor for inadequate glycemic control (37). With longer duration of T2D, there is usually further deterioration of the function of the pancreas and increase in insulin resistance, which makes it more difficult to control blood glucose level. The longer duration of T2D may result into the complications, which could negatively affect glycemic control either directly through inflammation and disturbance of the body’s metabolism or indirectly through the effect of poly-pharmacy, anxiety, depression and stress (34, 37).

The findings of this study suggested that BMI has no significant association with medication adherence and glycemic control. On the contrary, many previous studies reported that BMI was significantly associated with medication adherence (48, 49) and glycemic control (36, 42, 50). It was reported that weight reducing program improves the quality of life of obese patients with T2D (51). Preventing overweight is always recommended for patients with T2D regardless of their treatment status. Healthcare providers should thus continue to emphasize encouraging patients with T2D to maintain a healthy weight. Our findings in the present study revealed that medication adherence was not significantly associated with glycemic control, which was consistent with prior studies (50, 52, 53) However, a number of studies have shown that better medication adherence was related to improved glycemic control for patients with T2D (11, 27, 37, 42, 47). The possible reason is that non-adherence to medication affects the control of dysglycemia on a day-to-day basis and therefore results in inadequate glycemic control (27). Further research is needed to comprehensively explore the association between medication adherence and glycemic control in patients with T2D.

The results of this study may help healthcare providers realized that evaluating beliefs about medicines might critical for recognizing patients at risk of medication non-adherence and inadequate glycemic control. Because of the relatively low resource requirements and ease of implementation, assessing patients’ medication beliefs must be routinely practiced in diabetes management in our region. Previous studies revealed a positive correlation between medication literacy and medication beliefs, and demonstrated that enhancing medication literacy can positively impact medication beliefs, alleviate concerns, and ultimately improve medication adherence and health outcomes (53, 54). Pharmacist-led intervention was effective in optimizing medication adherence and improving glycemic control among diabetic patients (19, 49, 55). By participating in the clinical teams, pharmacists and nurses can offer an additional opportunity for structured counseling and motivational interviewing, or share decision-making models aimed at modifying patients’ medication beliefs.

### Strengths and limitations

This study clearly revealed the current status of suboptimal glycemic control and medication adherence among patients with T2D in Xi’an, China. Notably, this was one of the few studies examining beliefs about medicines among diabetes patients in Northwestern China, addressing a significant research gap in this population. The large sample size, multi-hospital design, use of validated instruments, and comprehensive statistical analysis strengthened the study’s reliability. All 3 glycemic indexes - FPG, PPG, and HbA1c levels - were obtained, which provided a comprehensive estimation of diabetes control in these patients. This study provided valuable insights into diabetes management for similar healthcare settings. There are several study limitations. First, the sample was collected from two large tertiary hospitals in Xi’an, and the results may not fully be extrapolated to the population with T2D in other regions. The hospital-based sample used in this study may limit the generalizability of the findings to community settings. Further research with more diverse samples will be considered to expand the generalizability of these findings. Second, a self-reported patient questionnaire was used to measure medication adherence, which was relatively easy but might be influenced by recall bias or social desirability bias. Thirdly, the absence of key behavioral and psychosocial factors - such as diet, depression, health literacy, and social support - limits the interpretation of adherence and glycemic outcomes. Fourthly, because there was no local adherence rate data available in Xi’an City, the assumption of 50% medication adherence was adopted in this study, which was one of the limitations of this study. Last but not least, the cross-sectional design precludes any causal inference. The association between injectable therapies and inadequate glycemic control without distinguishing between disease severity.

## Conclusion

The overall medication adherence among patients with T2D who were treated was 32.5%, while 30.5% of patients achieved adequate glycemic control in Xi’an. Findings of the present study provided evidence that medication beliefs are important determinants of medication adherence and glycemic control. Pharmacists and nurses could play an important role in educational and behavioral intervention programs on patients’ beliefs about medicines.

## Data Availability

The original contributions presented in the study are included in the article/**Supplementary Material**. Further inquiries can be directed to the corresponding authors.
