# Supplementary material for: The association of beliefs about medicines with medication adherence and glycemic control among patients with type 2 diabetes: a cross-sectional survey
Source: Front Endocrinol (Lausanne). 2026 Feb 10;17:1691701. doi: 10.3389/fendo.2026.1691701 (PMC12929140; doi:10.3389/fendo.2026.1691701)
Supplement: Supplementary file 3 [file DataSheet3.docx]

Hello! These are statements we have made about the hypoglycemic agents (oral or injectable agents) prescribed for you. Please truthfully fill in the following information and tick the appropriate option that best suits your situation. There are no right or wrong answers. We are interested in your personal views. Your answer will be kept confidential and will not have any impact on your treatment.

**Age (years):**

**Gender:** A. Female B. male

**Height (m):**

**Weight (kg):**

**Smoking status:**  A. No B. Yes

**Alcohol consumption:**  A. No B. Yes

**Education level:**  A. ≤ High school

B. >High school

**Employment status：**A. Not employed B. Employed

**Income/month:**  A. <4000 yuan B. ≥4000 yuan

**How long have you been diagnosed with type 2 diabetes?**

A. <5 years B. 5-10 years C. >10 years

**Regular self-monitoring of blood glucose:**  A. No B. Yes

**ADHERENCE TO REFILLS AND MEDICATIONS SCALE (ARMS-D)**

It is common for people to miss taking the hypoglycemic agents (oral or injectable agents) from time to time, or to take it differently than prescribed. I would like to ask you about how you actually take your hypoglycemic agents. There are no right or wrong answers. For each question, please answer “none of the time,” “some of the time,” “most of the time,” or “all of the time.”

1. How often do you forget to take your medicine?

A. None B. Some C. Most D. All

1. How often do you decide not to take your medicine?

A. None B. Some C. Most D. All

1. How often do you forget to get prescriptions filled?

A. None B. Some C. Most D. All

1. How often do you run out of medicine?

A. None B. Some C. Most D. All

1. How often do you skip a dose of your medicine before you go to the doctor?

A. None B. Some C. Most D. All

1. How often do you miss taking your medicine when you feel better?

A. None B. Some C. Most D. All

1. How often do you miss taking your medicine when you feel sick?

A. None B. Some C. Most D. All

1. How often do you miss taking your medicine when you are careless?

A. None B. Some C. Most D. All

1. How often do you forget to take your medicine when you are supposed to take it

more than once a day?

A. None B. Some C. Most D. All

1. How often do you put off refilling your medicines because they cost too much money?

A. None B. Some C. Most D. All

1. How often do you plan ahead and refill your medicines before they run out?

A. None B. Some C. Most D. All

**Beliefs about Medicines Questionnaire-Specific (BMQ-Specific)**

We would like to ask you about your personal views about the hypoglycemic agents prescribed for you. Please show how much you agree or disagree with them by ticking the appropriate option. There are no right or wrong answers. We are interested in your personal views.

1. My health, at present, depends on my medicines.

A. strongly agree B. agree C. uncertain D. disagree E. strongly disagree

1. Having to take medicines worries me.

A. strongly agree B. agree C. uncertain D. disagree E. strongly disagree

1. My life would be impossible without my medicines.

A. strongly agree B. agree C. uncertain D. disagree E. strongly disagree

1. I sometimes worry about long-term effects of my medicines.

A. strongly agree B. agree C. uncertain D. disagree E. strongly disagree

1. Without my medicines I would be very ill.

A. strongly agree B. agree C. uncertain D. disagree E. strongly disagree

1. My medicines are a mystery to me.

A. strongly agree B. agree C. uncertain D. disagree E. strongly disagree

1. My health in the future will depend on my medicines.

A. strongly agree B. agree C. uncertain D. disagree E. strongly disagree

1. My medicines disrupt my life.

A. strongly agree B. agree C. uncertain D. disagree E. strongly disagree

1. I sometimes worry about becoming too dependent on my medicines.

A. strongly agree B. agree C. uncertain D. disagree E. strongly disagree

1. My medicines protect me from becoming worse.

A. strongly agree B. agree C. uncertain D. disagree E. strongly disagree

1. These medicine give me unpleasant side effects.

A. strongly agree B. agree C. uncertain D. disagree E. strongly disagree
